# Suppressor of cytokine signaling-1 mimetic peptides attenuate lymphocyte activation in the MRL/lpr mouse autoimmune model

**DOI:** 10.1038/s41598-021-86017-4

**Published:** 2021-03-18

**Authors:** Jatin Sharma, Teresa D. Collins, Tracoyia Roach, Shiwangi Mishra, Brandon K. Lam, Zaynab Sidi Mohamed, Antia E. Veal, Timothy B. Polk, Amari Jones, Caleb Cornaby, Mohammed I. Haider, Leilani Zeumer-Spataro, Howard M. Johnson, Laurence M. Morel, Joseph Larkin

**Affiliations:** 1 Department of Microbiology & Cell Science, University of Florida, Museum Road Building 981, PO Box 110700, Gainesville, FL 32611 USA; 2 Department of Pathology, Immunology, and Laboratory Medicine, University of Florida, Gainesville, FL 32610 USA

**Keywords:** Cell biology, Immunology, Diseases, Molecular medicine, Pathogenesis, Rheumatology

## Abstract

Autoimmune diseases are driven largely by a pathogenic cytokine milieu produced by aberrantly activated lymphocytes. Many cytokines, including interferon gamma (IFN-γ), utilize the JAK/STAT pathway for signal propagation. Suppressor of Cytokine Signaling-1 (SOCS1) is an inducible, intracellular protein that regulates IFN-γ signaling by dampening JAK/STAT signaling. Using Fas deficient, MRL/MpJ-Fas^lpr^/J (MRL/lpr) mice, which develop lupus-like disease spontaneously, we tested the hypothesis that a peptide mimic of the SOCS1 kinase inhibitory region (SOCS1-KIR) would inhibit lymphocyte activation and modulate lupus-associated pathologies. Consistent with in vitro studies, SOCS1-KIR intraperitoneal administration reduced the frequency, activation, and cytokine production of memory CD8^+^ and CD4^+^ T lymphocytes within the peripheral blood, spleen, and lymph nodes. In addition, SOCS1-KIR administration reduced lymphadenopathy, severity of skin lesions, autoantibody production, and modestly reduced kidney pathology. On a cellular level, peritoneal SOCS1-KIR administration enhanced Foxp3 expression in total splenic and follicular regulatory T cells, reduced the effector memory/naïve T lymphocyte ratio for both CD4^+^ and CD8^+^ cells, and reduced the frequency of GL7^+^ germinal center enriched B cells. Together, these data show that SOCS1-KIR treatment reduced auto-reactive lymphocyte effector functions and suggest that therapeutic targeting of the SOCS1 pathway through peptide administration may have efficacy in mitigating autoimmune pathologies.

## Introduction

Autoimmune diseases such as systemic lupus erythematosus (SLE) are often debilitating, polygenic, and present with varied clinical manifestations^1^. It is thought that autoimmune diseases occur when pathogenic T and B lymphocytes grow refractive to tolerance mechanisms. Follicular and peripheral Foxp3^+^ Tregs limit effector functions elicited by follicular helper T lymphocytes and peripheral T lymphocytes respectively^2–4^. In the context of SLE, follicular helper T cells drive the activation of self-reactive germinal center B cells and the production of disease driving auto-antibodies, while aberrantly activated memory T lymphocytes produce enhanced levels of pro-inflammatory cytokines such as IFN-γ. In addition to promoting the isotype switching of pathogenic antibody producing B lymphocytes, the inflammatory environment produced by aberrant cytokine signaling also amplifies and perpetuates disease pathogenesis^5^. Cytokine signaling is largely dependent on the activation of the intracellular janus kinase (JAK) and signal transducers and activators of transcription (STAT) signal transduction pathways^6^. Given that defects in T lymphocyte activation, accumulation, responsiveness to cytokine environment, and elicitation of effector function have all been implicated in patients with autoimmunity, the identification of intracellular regulatory checkpoints for these processes could lead to the development of novel therapeutic targets.

The suppressor of cytokine signaling (SOCS) family of intracellular proteins is highly conserved between vertebrate species^7^ and is critically involved in the regulation of lymphocyte activation, differentiation, and elicitation of effector functions^8,9^. SOCS proteins are induced to limit the duration of the inflammatory signaling cascade by targeting the JAK/STAT pathway^6,10,11^. Deficiency in SOCS1, a member of the SOCS protein family, has been implicated in lupus, scleritis, and asthma patients, suggesting a role of SOCS1 in the regulation of immune homeostasis^12–18^. The importance of SOCS1 in the prevention of pathogenic inflammation is underscored by the 100% perinatal lethality experienced by SOCS1 deficient (SOCS1^−/−^) mice^8,19^. SOCS1 functions through two important mechanisms: (1) SOCS1 possesses an N-terminal kinase inhibitory region (KIR) that inhibits the kinase activity of JAK2, JAK1, and TYK2, and the TLR4 adapter Mal (Myd88-adapter like) through competitive inhibition; and (2) SOCS1 contains a C-terminal SOCS box that promotes proteasomal degradation of intracellular intermediary proteins necessary for propagating cytokine-mediated inflammatory cascades^20–26^. The importance of the KIR region of SOCS1 has been validated through the increased survival of SOCS1^−/−^ mice made transgenic to express the KIR region^27^. In addition, we have previously demonstrated that a peptide mimic of the KIR region of SOCS1 (SOCS1-KIR) prolonged the survival of SOCS1^−/−^ mice, ameliorated pathology in a rodent model of multiple sclerosis, and mitigated experimental rodent uveitis^12,14,28,29^. Importantly, SOCS1 and SOCS1-KIR have been associated with reducing Foxp3^+^ Treg plasticity under inflammatory conditions^12,30,31^.

MRL/lpr mice possess a defect in the Fas gene which promotes the dysregulated activation and accumulation of T and B lymphocytes, and serves as a pertinent, though aggressive, model to study lymphocyte mediated autoimmunity^32–35^. The defective Fas gene-mediated decreased functionality of regulatory T cells, accumulation of Th1 biased lymphocytes, and accumulation of anti-nucleic acid antibody-producing B lymphocytes drive an aggressive autoimmune phenotype relevant to many human autoimmune diseases including lupus, Sjogren’s syndrome, arthritis, and autoimmune lymphoproliferative syndrome (ALPS)^32–35^. In this study, we tested the hypotheses that intraperitoneal administration of SOCS1-KIR will: (1) modulate regulatory T lymphocyte populations, (2) attenuate lymphocyte activation, and (3) therapeutically mitigate autoimmune pathology in the MRL/lpr mouse. We show that SOCS1-KIR administration diminished the accumulation of IFN-γ-producing memory T cells, lymphadenopathy, and cutaneous disease. On a cellular level, SOCS1-KIR administration enhanced Foxp3 expression in both total splenic Tregs and follicular Tregs (Tfr). In addition, SOCS1-KIR treatment reduced the frequency of GL7^+^ cells, which are enriched for germinal center B cells^36^. These data suggest that therapeutic targeting of the SOCS1 pathway, through the use of peptides that mimic SOCS1 activity, may serve as a novel approach for the treatment of lymphocyte mediated autoimmunity.

## Materials and methods

### Mice

Female MRL/MpJ-Fas^lpr^/J (MRL/lpr) mice were acquired from the Jackson Laboratory (Bar Harbor, ME) and housed in specific pathogen free conditions at the University of Florida Cancer and Genetics Animal Care Facility, in strict accordance of approved protocols by the Institutional Animal Care and Use Committee-accredited Association of Assessment and Accreditation for Laboratory Animal Care, and in accordance to ARRIVE guidelines.

### Peptide synthesis

The SOCS1-KIR mimetic peptide (^53^DTHFRTFRSHSDYRRI), SOCS1-KIR dimeric variant (DTHFRTFRSHSDYRRIGGGGGDTHFRTFRSHSDYRRI), or pJAK2 (^1001^LPQDKEYYKVKEP) was generated in-house using Applied Biosystems 431a automated peptide synthesizer (Applied Biosystems, Carlsbad, CA) by conventional fluorenylmethylcarbonyl chemical methods as described^37,38^, or purchased from GenScript (Piscataway, NJ) at 95% purity. A palmitoyl-lysine (a lipophilic group) was added to the N-terminus of the peptides during the final step to assist in cell penetration. Peptides were characterized by high-performance liquid chromatography (HPLC) and mass spectrometry. Peptides were dissolved dropwise in DMSO (Thermo Scientific, Rockford, IL), then suspended to final administration volume in sterile PBS (Sigma Aldrich St. Louis, MO), or dissolved in Barnstead Nanopure water prior to use.

### Animal treatments

MRL/lpr mice, aged 8 (cohort 1) or 12 (cohort 2) weeks, were randomized through computer algorithm before receiving intraperitoneal injections of SOCS1-KIR peptide (10 μg/g animal weight) or PBS carrier 3 times per week. Treatments began 1 week after animal facility acclimation and extended through the end of experiments at 26 weeks, or animal sacrifice due to morbidity. For cohort 3, 8-week-old MRL/lpr mice received intraperitoneal injections of SOCS1-KIR peptide (60 μg/animal), SOCS1-KIR dimer (60 μg/animal), or carrier once daily. Treatments began 1 week after animal facility acclimation and extended through the end of experiments at 15 weeks. Mice were weighed weekly and general health evaluated using the standard body score index. Onset of lymphadenopathy was assessed based on a caliper scoring system from 1 to 4 where the following values were assigned: (1) No lymphadenopathy, (2) lymphadenopathy at multiple sites (< 2 cm diameter), (3) lymphadenopathy at multiple sites (> 2 cm diameter), (4) decline in health requiring euthanasia. Skin lesion severity was based on a scale of 0–4 (0 = no lesion, 1 = mild, 2 = moderate, 3 = clearly visible, 4 = severe).

### Flow cytometry

Blood samples were clotted on ice, then cells were centrifuged (13.3 K RPM for 10 min at room temperature) and supernatant was removed. Pellets were then lysed with 1 ml of RBC lysis buffer (Sigma Aldrich, St. Louis, MO) for 5 min. After a second centrifugation, supernatant was removed and samples were transferred to v-bottom flow plates using 100 μl of media (Greiner Bio-One GmbH, Kremsmuenster, Austria). Single-cell suspensions of peripheral (superficial cervical, axillary, mediastinal, brachial, and inguinal) lymph nodes (LN) and/or spleen were cultured under varied conditions as indicated in text. For cohort 1 & 2, cells were stained with the following monoclonal antibodies for flow cytometric analysis: anti-CD4-Pacific Blue (RM4-5; BD Pharmingen, San Diego, CA), -APC, -PE, and -FITC (GK1.5; eBioscience, San Diego, CA); anti-CD8α-Alexa Fluor 700 (RPA-T8), -FITC and -PE (53-6.7; BD Pharmingen); anti-CD25-APC (PC61.5; BD Pharmingen), -PE and -Alexa Fluor 700 (53-6.7; eBioscience); anti-CD44-PE (IM7; BioLegend, San Diego, CA); anti-CD69-APC and -PE (H1.2F3; BioLegend); anti-IFN-γ-APC, and -PE (XMG1.2; eBioscience) and -FITC (XMG1.2; BioLegend); anti-CD3-PB (500A2) and APC (145-2c11). In cohort 3, the following antibodies were utilized as described in^39^: anti-CD4-PB (RAM4-5), anti-CXCR5-Biotin (2G8) + Streptavidin-PerCP, PHA-L-FITC, anti-CD25-AF700 (PC61.5), anti-BCL6-APC (K112-91), Viability dye-APC-Cy7, anti-Foxp3-PE (FJK-16s), anti-CD3-BV711 (145-2C11), anti-CD8-PerCPCy5 (53-6.7), anti-PD1-PECy7 (RMP1-30), anti-CD44-V500 (IM7), anti-CD62L-PE (MEL-14), anti-CD19-PECy7 (ID3), anti-IgD Biotin (217-170) + Streptavidin-PerCP, anti-Ly77-Pacific blue (GL7), anti-B220-BV510 (RA3-6B2), and anti-CD138-APC (281-2). All antibodies were purchased from BDBiosciences or eBioscienes, unless otherwise indicated. Intracellular expression of IFN-γ was measured after cell activation as previously described^40^. Briefly, cells were incubated in a cocktail of PMA, ionomycin, and brefeldin A for 4 h, then fixed in a 2% paraformaldehyde solution. A total of 50,000 live events were collected by LSRII (BD Bioscience). All flow cytometry analysis was performed using FlowJo v10 (Tree Star, San Carlos, CA).

### RNA isolation and RT-qPCR

Total RNA was extracted as previously described^41^ from both blood derived cells and lymph nodes of age-matched MRL/lpr mice. First-strand cDNA synthesis was completed using iScript Kit (Bio-Rad). iQ SYBR Green Supermix (Bio-Rad) and gene-specific primers (Table 1) were utilized to amplify relative amounts of cDNA on a CFX Connect Real Time System (Bio-Rad). The fold change expression was calculated using the value 2^−∆∆CT^ method, with Bio-Rad software and *Gapdh* as the reference gene.

**Table 1:** List of primers used in the study.

Gene	Forward primer	Reverse primer
IFN‐γ	5′‐AAC TAT TTT AAC TCA AGT GGC AT‐3′	5′‐AGG TGT GAT TCA ATG ACG‐3′
MHC‐II (H2‐Aα‐chain)	5′‐AAG AAG GAG ACT GTC TGG ATG C‐3′	5′‐TGA ATG ATG AAG ATG GTG CCC‐3′
Β‐actin	5′‐GAT CTG GCA CCA CAC CTT CT‐3′	5′‐GGG GTG TTG AAG GTC TCA AA‐3′

### Cytokine secretion analysis and ELISAs

A total of 1–2 × 10^6^ cells isolated from lymph nodes were plated in complete culture medium (Dulbecco’s Modified Eagle Medium (DMEM), 10% FBS, 1% antibiotic–antimycotic (ABAM), and 0.1% beta-mercaptonol) with or without 3 μg/ml anti-CD3 and 3 μg/ml anti-CD28 antibodies (BD Biosciences, San Diego, CA), 20 μM SOCS1-KIR, 20 μM SOCS1-KIR Dimer, and 20 μM pJAK2 mimetic peptides. IFN-γ was measured with an ELISA kit (BD Pharmingen) as previously described^41^ on supernatants harvested after a 5 days incubation.

Serum was collected from animals weekly to assess the production of anti-dsDNA IgG. Anti-dsDNA IgG was measured in sera diluted 1:1000, 1:3000, or 1:9000 in plates coated with 50 μg/mL dsDNA. Bound IgG was detected using alkaline phosphatase-conjugated anti-mouse IgG diluted 1:1000. The absorbance at 450 nm was measured.

### Renal pathology

Kidneys were embedded in paraffin in week 15. Paraffin-embedded kidney samples were sectioned and stained with hematoxylin and eosin (H&E). Digitized H&E images were analyzed using Aperio ImageScope as previously described^42^. The area was measured in at least 40 glomeruli per sample by blinded reviewers. Kidney pathology was scored and measured on a semi-quantitative scale of 0–3+ as previously described^43^.

### Statistical analysis

GraphPad Prism v8 was used to calculate statistical significance between various groups using Student’s *t*-test and ANOVA coupled with Dunnett’s or Sidak’s multiple comparison tests. p values ≤ 0.05 was considered significant as indicated within each figure.

## Results

### SOCS1-KIR mimetic peptides mitigate MRL/lpr T lymphocyte activation and cytokine production in vitro

**Figure 1:**
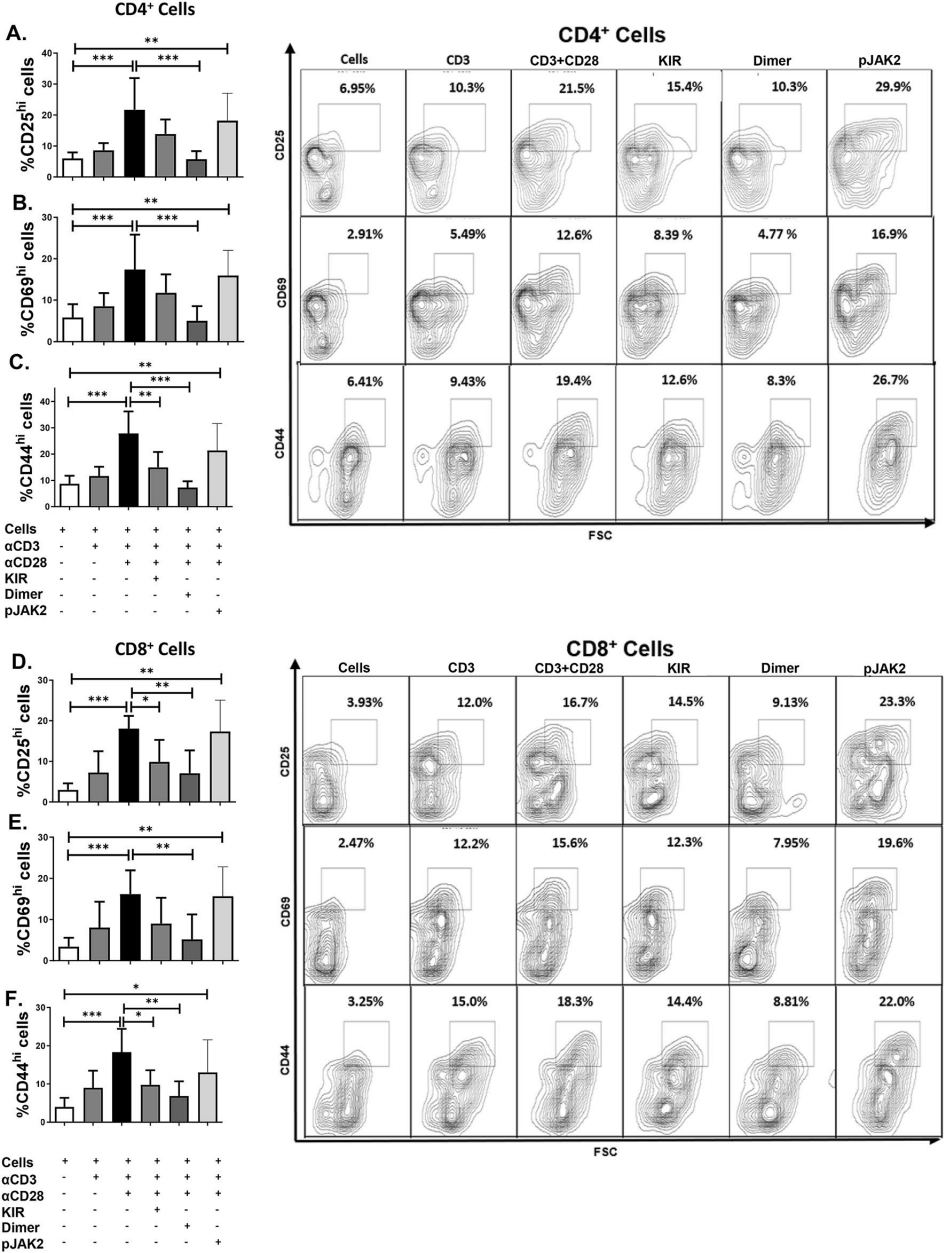
SOCS1-KIR mimetic peptides mitigate MRL/lpr T lymphocyte activation in vitro. Single cell suspensions of pooled MRL/lpr lymph nodes were stimulated with anti-CD3/anti-CD28 in the presence or absence of SOCS1-KIR monomer mimetic, SOCS1-KIR dimer mimetic, or pJAK2 peptides for 5 days. (**A**–**F**) Quantification (left) of representative flow cytometric analysis (right) depicting changes in CD25, CD69, and CD44 surface expression on MRL/lpr T lymphocytes. Data are from two independent experiments, with 4 animals per group (graphs display average frequency, error bars, SD). *p < 0.05, **p < 0.01, and ***p < 0.001 (One-way ANOVA with Dunnett’s multiple comparison test). The figure was prepared in Graphpad prism v9 [https://www.graphpad.com/scientific-software/prism/].

To assess the ability of SOCS1 mimetic peptides (SOCS1-KIR) to modulate T lymphocyte activation, we cultured a single cell suspension from total axillary, brachial, cervical, and inguinal LN isolated from 8-week old MRL/lpr mice with anti-CD3 or anti-CD3/anti-CD28 antibodies, in the presence of SOCS1-KIR monomeric or a dimeric peptide variants (SOCS1-KIR dimer) for 5 days. As a control, activated cultures were also incubated with a peptide corresponding to the region of JAK2 previously shown to interact with endogenous SOCS1 thereby acting as a SOCS1 antagonist (pJAK2 1001–1013)^44^. anti-CD3 stimulation yielded a modest upregulation of CD25, CD69, and CD44 in CD4^+^ (Fig. 1A–C) and CD8^+^ (Fig. 1D–F) T lymphocytes. As expected, anti-CD3/anti-CD28 co-stimulation yielded twofold to fivefold increases in the frequency of the highly activated CD4^+^ and CD8^+^ lymphocytes bearing increased size (based on FSC) and elevated surface levels of CD25, CD69, and CD44 when compared to unstimulated cells. The co-incubation with the SOCS1-KIR dimer reduced all three of these activation markers, while SOCS1-KIR reduced the frequency of CD4^+^ and CD8^+^ T lymphocytes bearing high surface expression of CD44 and the frequency of CD25^+^CD8^+^ T lymphocytes. Conversely, co-incubation with pJAK2 (1001–1013) failed to reduce activation.

**Figure 2:**
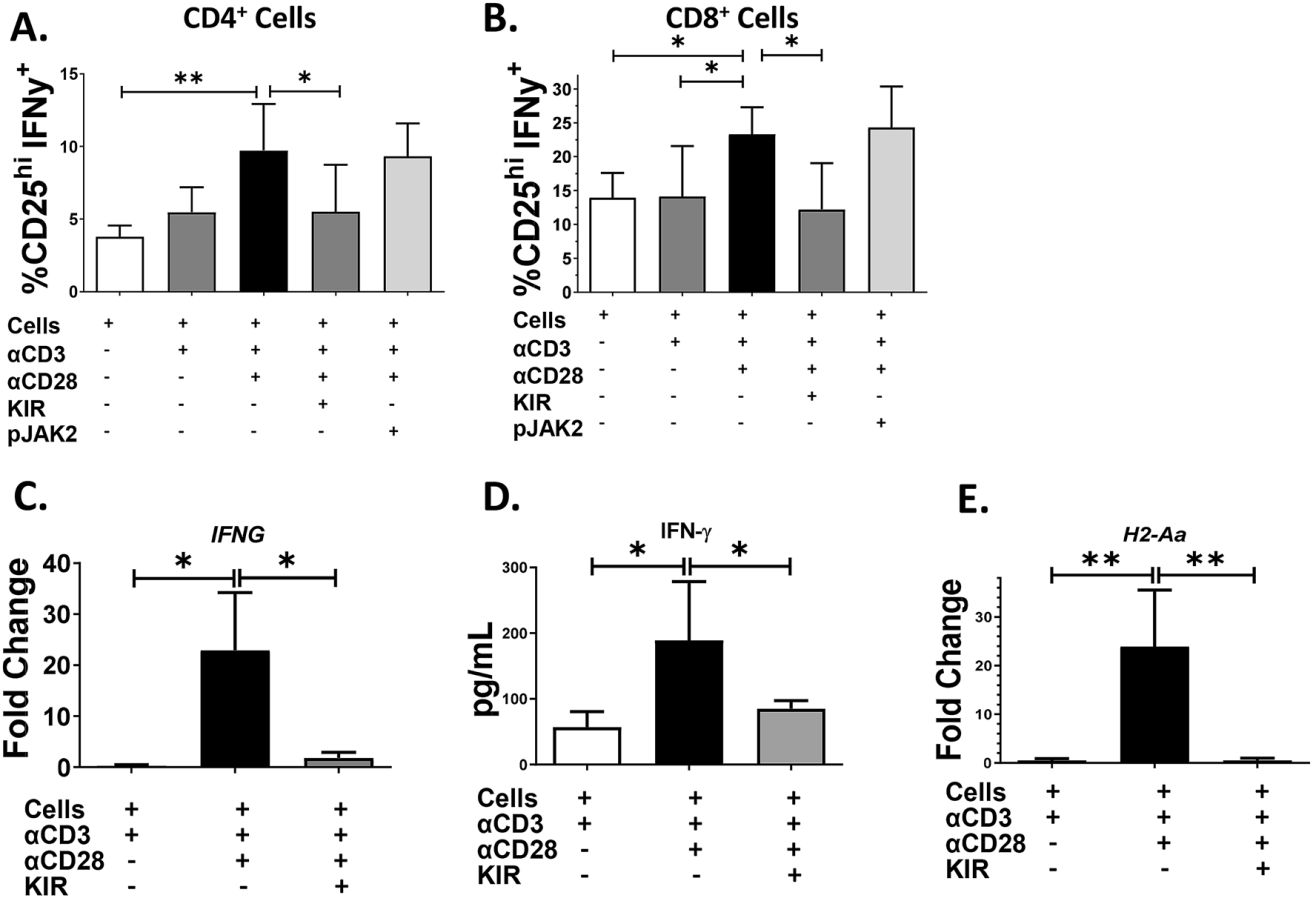
SOCS1-KIR treatment reduces interferon gamma production by activated T cell subsets. LN and spleen were isolated from MRL/lpr mice and cultured for 5 days under indicated conditions. (**A**,**B**) Quantification of flow cytometric analysis. Cell populations were gated for the co-expression of CD4 or CD8 with CD25 and IFN-γ. (**C**) Fold change of *ifng* mRNA expression relative to *GAPDH* after 5 days of culture. (**D**) IFN-γ ELISA performed on supernatants after cell culture for 5 days. (**E**) Fold change of *H2-Aa* mRNA expression relative to *GAPDH* after culture for 5 days. The results were compiled from two independent experiments with 3–5 animals per treatment group. RT-qPCR and ELISA assays were performed in triplicate (error bars, SD). *p < 0.05, **p < 0.01, and ***p < 0.001 (One-way ANOVA with Dunnett’s multiple comparison test). The figure was prepared in Graphpad prism v9 [https://www.graphpad.com/scientific-software/prism/].

Since IFN-γ production is associated with lupus progression^45–48^ and it is modulated by endogenous SOCS1^12,49,50^, we next assessed IFN-γ production following incubation with SOCS1 mimetic or antagonist peptides. Remarkably, co-incubation with SOCS1 mimetic, but not pJAK2 (1001–1013), significantly reduced the frequency of CD4^+^ and CD8^+^ CD25^+^ IFN-γ^+^ T lymphocytes (Fig. 2A,B). The SOCS1-KIR mimetic also reduced IFN-γ mRNA expression and protein secretion in LN cells cultured in these conditions (Fig. 2C,D). Consistent with MHC class II upregulation by IFN-γ, *H2-Aa* expression levels were significantly reduced in stimulatory conditions co-cultured with SOCS1-KIR, compared to stimulatory conditions alone (Fig. 2E). In summary, in vitro administration of SOCS1-KIR to MRL/lpr leukocytes inhibited T lymphocyte activation and production of IFN-γ.

### Peptide administration decreases the frequency of memory T lymphocytes within peripheral blood and secondary lymphoid organs, while decreasing splenomegaly and lymphadenopathy

**Figure 3:**
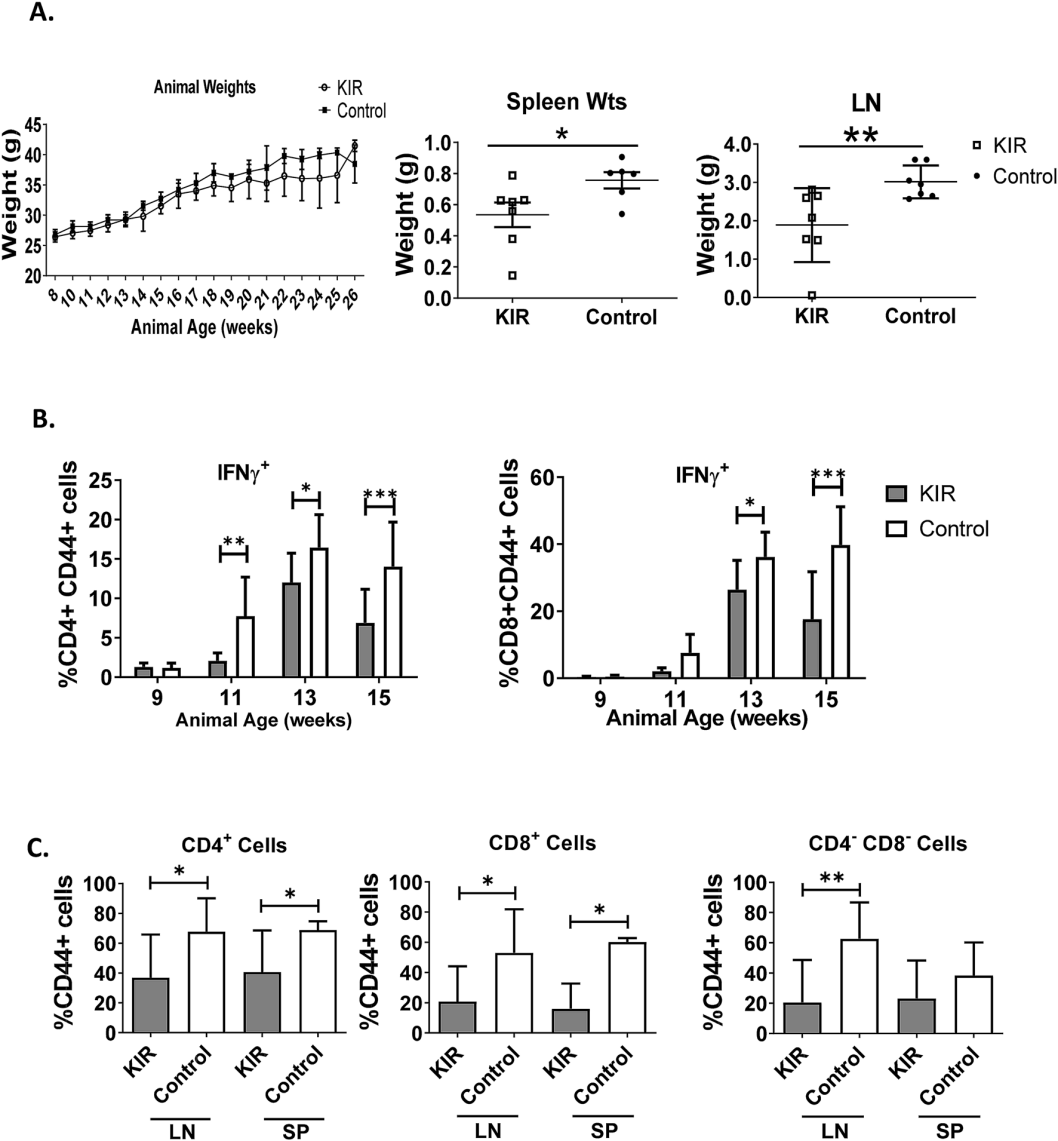
Peptide administration decreases the frequency of memory T lymphocytes within peripheral blood and secondary lymphoid organs, while decreasing splenomegaly and lymphadenopathy in vivo. (**A**) Line graph displaying weight of animals throughout the study (left). Total weight of lymph nodes and spleens extracted from treated and untreated animals at sacrifice (middle, right). Each symbol represents an individual mouse. (**B**) Bar graph showing the percentage of CD4^+^ and CD8^+^ cells that are double positive for CD44 and IFN-γ within peripheral blood of SOCS1-KIR treated and untreated mice over time. (**C**) Bar graphs showing the frequency of CD4^+^, CD8^+^, or CD4^−^CD8^−^ that are also CD44^+^ cells within the LN and spleen of treated or untreated mice at 15 weeks. Data are from two independent experiments with four animals per group (error bars, SD). *p < 0.05, **p < 0.01, and ***p < 0.001 (two-way ANOVA with sidak’s multiple comparison test (**A**) and unpaired student’s t-test (**B**,**C**)). The figure was prepared in Graphpad prism v9 [https://www.graphpad.com/].

MRL/lpr mice experience pronounced lympho-accumulation that often requires euthanasia. Using a cohort of four SOCS1-KIR treated and four PBS control 8-week-old MRL/lpr mice, we initially assessed the effect of the SOCS1 mimetic peptide on lymphadenopathy. 75% of the PBS treated animals in experimental cohort 1 required premature euthanasia after 20 weeks compared to only 25% in the SOCS1-KIR treated group (Figure S1). This result provided insight into the possible efficacy of SOCS1-KIR in the MRL/lpr mouse model. We next assessed SOCS1-KIR mitigation of lymphadenopathy using a larger sample size. Although SOCS1-KIR administration did not affect the overall weight of the mice, the lymph nodes and spleens from SOCS1-KIR treated animals were significantly smaller (Fig. 3A). Together, these results suggested that SOCS1-KIR treatment may reduce lymphadenopathy and splenomegaly in the MRL/lpr mouse.

Given that dysregulated IFN-γ production by memory T cells facilitates disease pathogenesis in MRL/lpr mice^51^, and that endogenous SOCS1 inhibits IFN-γ production (^12,49,50^ and our in vitro results), we hypothesized that SOCS1-KIR peptide administration could inhibit the presence of IFN-γ-producing memory T lymphocytes (CD44^+^) within the peripheral blood of MRL/lpr mice. At 9 weeks of age, there was a negligible amount of CD44^+^ IFN-γ^+^ T lymphocytes within the peripheral blood of PBS treated (control) MRL/lpr mice that markedly increased over the next 7 weeks (Fig. 3B). In contrast, the frequency of CD44^+^ IFN-γ^+^ CD4^+^ and CD8^+^ T lymphocytes within the peripheral blood of SOCS1-KIR treated mice were significantly reduced in comparison to control mice over the same time period (Fig. 3B and Figure S2). At sacrifice, the frequency of CD44^+^ cells was reduced in both CD4^+^ and CD8^+^ T lymphocyte populations in the spleen and LN of mice treated with SOCS1-KIR, while the frequency of CD44^+^ cells in CD4^−^ CD8^−^ double negative T cells (often associated with SLE and ALPS progression) was reduced only in lymph nodes (Fig. 3C). Together, these data show that SOCS1-KIR treatment reduced cellular accumulation within the secondary lymphoid organs of MRL/lpr mice, possibly through the reduction of memory T cells.

### SOCS-1 mimetic peptides reduce spontaneous skin lesions, anti-dsDNA IgG, and glomerular enlargement in MRL/lpr mice

**Figure 4:**
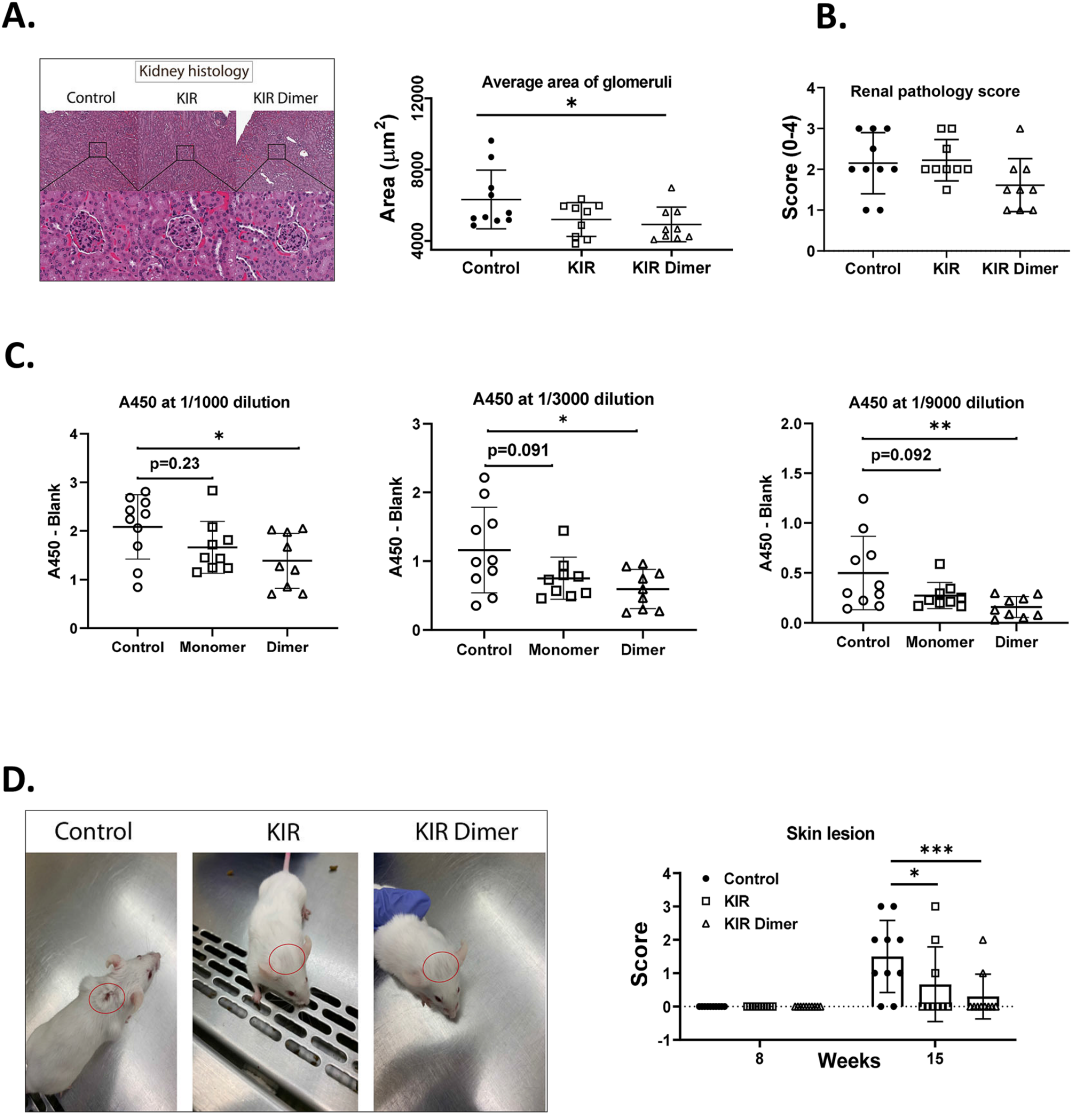
Reduction of lupus associated kidney pathologies, anti-DNA IgG, and skin lesions by treatment with the mimetic peptide. (**A**) Average glomeruli area measured in kidney H&E sections. Representative images on week 15 are provided (left). (**B**) Renal pathology score marked on a semi-quantitative scale of 0–3+ . (**C**) Submandibular bleeds were collected on week 15 and assayed for anti-dsDNA IgG at 1/1000, 1/3000, and 1/9000 sera dilution. (**D**) Skin lesions scored on a scale of 0–4 [0 = None, 1 = mild, 2 = moderate, 3 = clearly visible, 4 = severe]. Representative images on week 15 are provided (left). Data are from one independent experiment with 9–10 mice per group (error bars, SD).*p < 0.05, **p < 0.01, ***p < 0.001 (one-way ANOVA with dunnett’s multiple comparison test). All the graphs were prepared in Graphpad prism v9 [https://www.graphpad.com/scientific-software/prism/]. The representative images were edited using Adobe Illustrator v25.2 [https://www.adobe.com/products/illustrator.html.

We next examined the ability of the peptide to regulate spontaneous lesions and antibody production in MRL/lpr mice. Monomeric SOCS1-KIR, the dimeric variant SOCS1-KIR dimer (previously shown to be superior in vitro in Fig. 1), or PBS-carrier were administered at 8-weeks of age until sacrifice at week 15. Although there was no statistical difference in renal pathology scores (Fig. 4B), there was a significant reduction in glomerulus size, which is another indication of kidney damage^42^ (Fig. 4A). There was also a reduction in the level of serum anti dsDNA IgG (Fig. 4C) that was present in SOCS1-KIR dimer treated mice. Finally, as can be seen in Fig. 4D, SOCS1-KIR mimetic peptide treated mice had reduced severity in spontaneous skin lesions (Fig. 4D). Together, these results suggest that animals receiving SOCS1-KIR treatments had reduced immune-mediated pathology.

### SOCS1 KIR and KIR dimer treatment increases Foxp3 expression in Tregs and follicular Tregs

**Figure 5:**
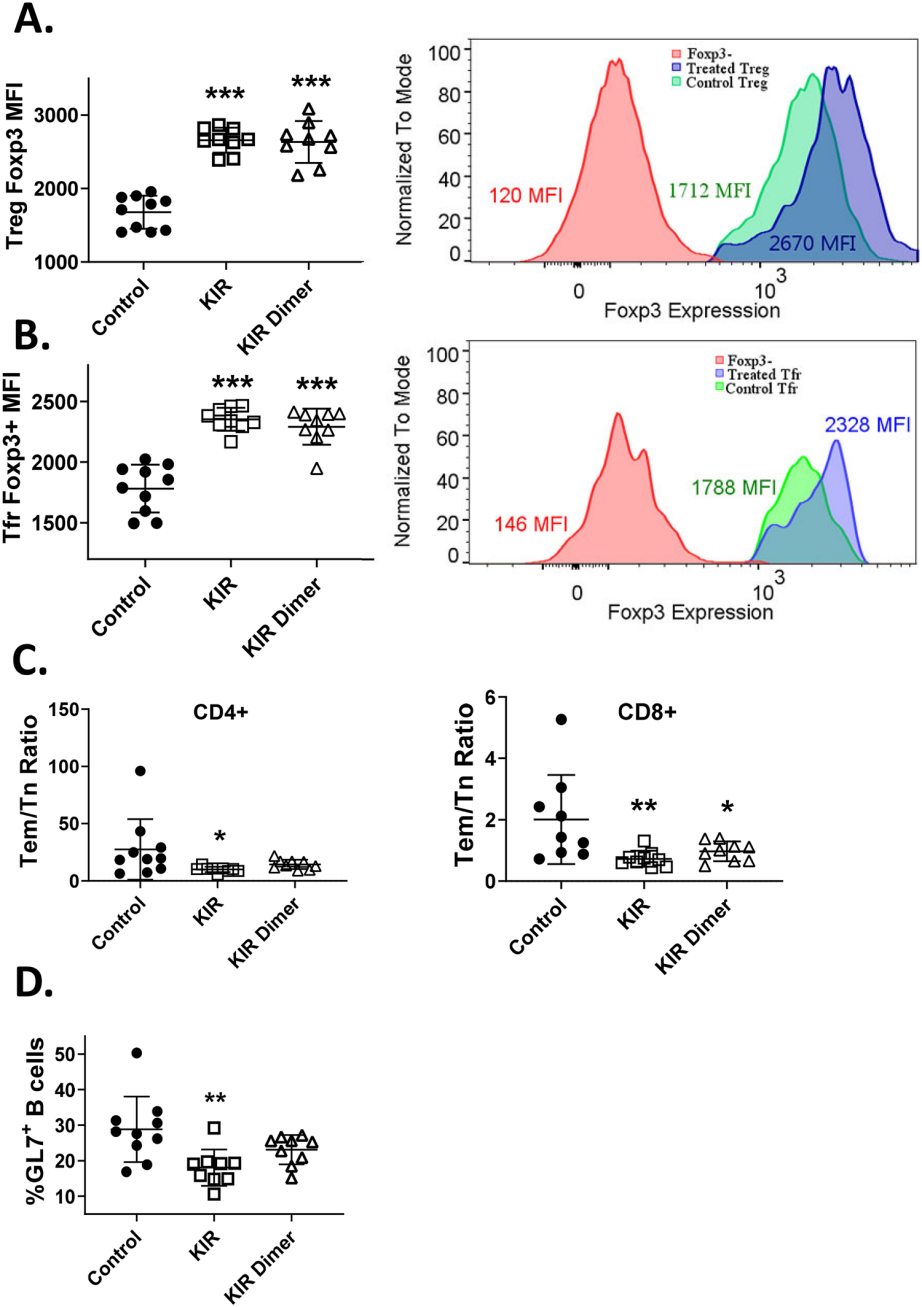
SOCS1 KIR mimetic peptide treatment enhances Foxp3 expression in regulatory T cells, attenuates T cell activation, and reduces germinal center B cells in vivo. (**A**) CD4^+^ Foxp3^+^ T regulatory cells and Foxp3 MFI. (**B**) CD4^+^ T follicular regulatory cells [CD4^+^ PD1^+^ CXCR5^+^ Bcl6^+^ Foxp3^+^] were analyzed by flow cytometry. (**C**) Ratio of CD4^+^ and CD8^+^ effector memory (CD44^+^ CD62L^−^) to naïve T cells (CD44^−^ CD62L^+^). (**D**) Frequency of Germinal Center B cells (CD19^+^ GL7^+^) and CD80^+^ Plasma cells (CD19^−^ CD138^+^). Each cohort had 9–10 animals. (Error bars, SD) *p < 0.05, **p < 0.01, ***p < 0.001 (One-way ANOVA followed by dunnett’s multiple comparison test)]. The histograms were prepared in FlowJo v10 [https://www.flowjo.com/solutions/flowjo]. All other graphs were prepared in Graphpad prism v9 [https://www.graphpad.com/scientific-software/prism/].

Treg suppressive abilities directly correlate with Foxp3 expression^52^. While high Foxp3 expression is generally an indicator of a stable suppressive phenotype, reduced Foxp3 expression is associated with decreased suppressive function and autoimmunity^52^. As we have previously shown that partial rescue of SOCS1^−/−^ mouse perinatal lethality by SOCS1-KIR mimetic peptide administration was correlated to enhanced Treg function and an overall increase in Foxp3 expression in CD4^+^ T cells^12^, we next evaluated both total and follicular Tregs in the spleen of mice treated with SOCS1-KIR monomer, dimer, or control by flow cytometry. Using the gating scheme present in Figure S3, we found that while there was no difference in total numbers of conventional Tregs (CD4^+^ Foxp3^+^) or follicular Tregs (CD4^+^ PD1^+^ CXCR5^+^ Bcl6^+^ Foxp3^+^) (Fig. S4A), there was a greater than 50% and 30% increase in Foxp3 mean fluorescent intensity (MFI) in the total and follicular Treg populations, respectively (Fig. 5A,B). As previous studies have shown that SOCS1 is important in stabilizing Foxp3 and regulatory T cell function^12,30,31^, together these results suggest that the administration of SOCS-KIR peptide may serve to stabilize the phenotype of peripheral and follicular Tregs.

### SOCS1 KIR mimetic peptide treatment attenuates T cell activation and reduces CD19^+^GL7^+^ B cell populations enriched for germinal center B cells in vivo

In therapeutic cohort 1 we observed a reduction in CD44^+^ IFN-γ^+^ CD4^+^and CD44^+^ IFN-γ^+^ CD8^+^ memory T lymphocytes circulating in the blood of mice treated with the SOCS1-KIR monomer at 15 weeks of age (Fig. 3A), which corresponded to reduced memory CD4^+^ and CD8^+^ T cells within the spleen and lymph nodes (Fig. 3B). To better understand the immunomodulatory effects of SOCS1-KIR on T lymphocyte activation, we next evaluated changes within specific T lymphocyte populations present in the spleens of treated and control mice of cohort 3 at 15 weeks of age. Although total CD4^+^ and CD4^+^ effector memory cell populations were unaffected by mimetic treatments, the percentage of naïve CD4 T cells were consistently higher within SOCS1 mimetic peptide treated mice, reaching statistical significance in monomeric SOCS1-KIR treated mice (Figure S4B). In addition, monomeric SOCS1-KIR significantly reduced the CD4^+^ effector memory/naïve T lymphocyte ratio (Fig. 5C). The percentage and total number of naive splenic CD8^+^ T lymphocytes were increased by mimetic administration, reaching significance with the SOCS1-KIR dimeric peptide (Figure S4B). Indeed, in similar fashion to the CD4^+^ T lymphocytes, the CD8^+^ T effector memory/naïve cell ratio was significantly reduced by the mimetic peptide treatments (Fig. 5C). As we saw SOCS1-KIR mimetic peptide mediated reductions in antibody production, we next evaluated peptide mediated changes in splenic B cells. While many B cell subsets appeared unaffected (Figure S5), there was significant reduction (~ 40%) in the frequency of GL7^+^ B cells, which are enriched in germinal center B cells, within the SOCS1-KIR monomer treated group (Fig. 5D). This result aligns with two previous studies where tofacitinib, which also targets JAK/STAT signaling, was shown to reduce germinal center B cells post immunization in BALB/c mice and anti-dsDNA concentration in MRL/lpr mice respectively^53,54^. As the germinal center (GC) is the major source of high affinity class switched IgGs^36^, these data suggest that SOCS1-KIR regulation of GC B cell populations can in part explain lower anti-nucleic acid autoantibodies and reduced kidney-associated pathology in treated mice.

### SOCS1-KIR mediated lymphocyte changes correlated to reduced anti-DNA IgG production

**Figure 6:**
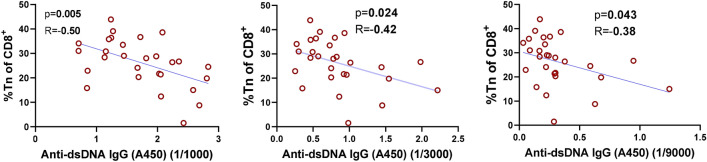
SOCS1-KIR mediated lymphocyte changes correlated to reduced anti-DNA IgG production. Linear correlation was evaluated for the relative frequency of CD8+ naïve T cells and anti-dsDNA IgG. Pearson’s correlation coefficient (R) and p values are indicated on the graphs. The figure was prepared in Graphpad prism v9 [https://www.graphpad.com/scientific-software/prism/].

Since anti-DNA IgG production is a common clinical immunopathology associated with lupus progression, we next analyzed the correlation between anti-DNA IgG production and SOCS1-KIR mediated leukocyte changes using the 1:1000, 1:3000, and 1:9000 serum dilutions. Foxp3 MFI in both total splenic and follicular Tregs trended towards a negative correlation with anti-dsDNA IgG production, reaching statistical significance at 1:3000 and 1:9000 serum dilutions, respectively (Supplemental Figure 6A,B). Notably, the frequency of naïve CD8^+^, but not CD4^+^ T lymphocytes—shown to be enhanced by SOCS1 peptide treatment (Fig. 6, supplemental Figure S6C), was also negatively correlated to anti-dsDNA IgG production at all serum dilutions. In addition, glomerular area trended towards positive correlation with anti-dsDNA IgG production, achieving statistical significance at the 1:9000 serum dilution (Supplemental Figure S6D), Together, these results suggest a clinical benefit was mediated by the SOCS1-KIR driven changes in T lymphocytes in vivo.

## Discussion

Although medical advances have significantly improved the quality of life in patients with autoimmune diseases, there remains a critical need for novel therapeutic options as many patients remain refractory or intolerant to existing strategies. The clinical manifestations observed in autoimmune diseases (such as SLE, ALPS, or Sjogrens syndrome) arise from defective tolerance mechanisms. In addition to regulatory T cells and tight regulation of pro-inflammatory cytokine/cytokine receptor levels, the suppressor of cytokine signaling family of proteins (SOCS) is also critically involved in the maintenance of self-tolerance^9,19^. While complete knockout of SOCS1 results in lethal auto-inflammatory disease, inadequate SOCS1 signaling is associated with lupus-like disease in several rodent models^50,55,56^. Recent studies have also associated variants in SOCS1 related genes, or defects in expression, with lupus and other auto-inflammatory diseases in humans^13,17,57–59^. Notably, the KIR of SOCS1 has a potent immuno-modulatory effect, made evident by the increased survival of SOCS1^−/−^ mice made transgenic to express KIR devoid of the SOCS box^27^. In this study, we show that administration of peptide variants of SOCS1-KIR to MRL/lpr mice mitigated skin lesions, lymphadenopathy, anti-dsDNA IgG levels, and reduced lupus associated kidney pathology. In addition, SOCS1-KIR administration enhanced Foxp3 expression in both total splenic Tregs and follicular Tregs, while reducing GL7^+^ B cells which are enriched for germinal center B cells.

Although clinical manifestations of SLE are largely driven by auto-antibody production by B lymphocytes, activated T lymphocytes are critical in SLE pathogenesis and a strong indicator of disease activity in patients^60–63^. CD4^+^, CD8^+^, and CD4^−^CD8^−^ T lymphocytes promote a pro-inflammatory environment conducive to lupus pathogenesis and generation of pathogenic anti-DNA Ig secreting B cells. Notably, interferon gamma, produced by CD4^+^ and CD8 effector T lymphocytes, plays an indispensable role in promoting the availability of auto-antigens and the development of TLR7 dependent autoreactive B cells that drive systemic autoimmunity^64–66^. In this study, we showed that SOCS1-KIR reduced the frequency anti-CD3/anti-CD28 activated, IFN-γ producing, CD4^+^ and CD8^+^ T lymphocytes from MRL/lpr mice in vitro. Moreover, in vivo administration of SOCS1-KIR reduced the frequencies of circulating and splenic T lymphocytes, and prevented the acquisition of the IFN-γ effector function. Notably, the peripheral CD4^+^ and CD8^+^ T lymphocytes in SOCS1-KIR treated mice were more biased towards a naïve phenotype compared to control MRL/lpr mice as indicated by the memory/naïve T cell ratio. These results could bear importance to human autoimmune diseases, such as SLE and ALPS, as the JAK/STAT pathway has been strongly implicated in driving T cell mediated immunopathogenesis^67–69^. Therefore, it is likely that one mechanism of action by SOCS1-KIR is the regulation of autoimmune-promoting effector functions by T lymphocytes. Future studies will further elucidate the mechanistic contribution of SOCS1-KIR to direct T cell activation, or indirect antigen presenting cell regulation.

The ability of follicular helper T cells to drive antibody production by B lymphocytes is tightly regulated by T follicular regulatory cells^3–5^. Accumulating evidence has implicated dysregulated follicular helper and regulatory T cell interactions in the generation of auto antibodies by B lymphocytes. We and others have shown that SOCS1 is critical for the stability of Foxp3^+^ Tregs under inflammatory conditions^12,30^, but a specific role in follicular regulatory T cells has not been explored to date. In this manuscript we shown that SOCS1 KIR treatment increased Foxp3 expression in both splenic and follicular Tregs, which is consistent with our previous studies showing that peritoneal administration of SOCS1-KIR can stabilize the Foxp3^+^ Treg population and facilitate the production of immunomodulatory cytokines^12,14,28^. We also observed decreased germinal center B cells and anti-dsDNA IgG production in mice treated with SOCS1-KIR mimetic peptides. Future work will assess the ability of SOCS1-KIR mimetic peptides to modulate marginal cell B cells, as they are also a relevant B cell population in MRL/lpr mice^70,71^. Notably, our renal results are supported by a previous paper which showed that peritoneal injection of SOCS1-KIR peptide abated renal pathology in a rodent model of type 2 diabetes^72^. The difference in effects between the monomer and dimeric variants could be due to increased valency of dimer while monomer being smaller and having better flexibility. However, further studies would have to be done to elaborate exact mechanistic differences between the peptide variants.

There has been reluctance to pursue therapeutic peptide use for the treatment of auto-inflammatory diseases by the pharmaceutical industry because of high degradation rates, poor target site delivery, and low stability^73^. However, therapeutic peptide use has been shown advantageous as they can be highly specific, safe, and well tolerated by humans. Peritoneal injected SOCS1-KIR has previously shown peak levels within the serum and plasma of injected animals around 6 h with effective clearance at 18 h^74^. The SOCS1-KIR mimetic peptide has a palmitoyl group facilitating intracellular delivery into target cells, thereby possessing a mechanism of action that is distinct to that of decoy receptors or antibodies that act extracellularly. It is therefore tempting to speculate that intracellular-targeting SOCS1-KIR peptides could be used in combination with other biologics or steroids, potentially lowering the effective dose and/or toxicity. This study extends our understanding of the use of SOCS mimetic peptides in the treatment of inflammatory disease by showing the regulation of lymphocytes and amelioration of lupus-associated pathologies. Our current study, combined with previous work, suggests that the use of peptide mimetics of SOCS proteins should be evaluated as a therapeutic strategy for the treatment of human autoimmune diseases.

## Supplementary information

Supplementary information.

## Abbreviations

ALPS: Autoimmune lymphoproliferative syndrome
CD: Cluster of differentiation
IFN: Interferon
JAK: Janus kinase
KIR: Kinase inhibitory region
LN: Lymph node
MHC: Major histocompatibility complex
SLE: Systemic lupus erythematosus
SOCS1: Suppressor of cytokine signaling
STAT: Signal transducer and activator of transcription
TLR: Toll-like receptor
Treg: Regulatory T cells
