# Supplementary material for: Suppressor of cytokine signaling-1 mimetic peptides attenuate lymphocyte activation in the MRL/lpr mouse autoimmune model
Source: Sci Rep. 2021 Mar 18;11:6354. doi: 10.1038/s41598-021-86017-4 (PMC7973732; doi:10.1038/s41598-021-86017-4)

Supplemental figures

Suppressor of cytokine signaling-1 mimetic peptides attenuate lymphocyte activation in the MRL/lpr mouse autoimmune model.

Jatin Sharma, [1,2] Teresa D. Collins,[1,2] Tracoyia Roach [3], Shiwangi Mishra,[1] Brandon K. Lam,[1] Zaynab Sidi Mohamed,[1] Antia E. Veal,[1] Timothy B. Polk[1], Amari Jones[1], Caleb Cornaby[3], Mohammed I. Haider,[1] Leilani Zeumer-Spataro[3], Howard M. Johnson,[1] Laurence M. Morel,[3] and Joseph Larkin, III[1*]

[1] *Department of Microbiology & Cell Science, University of Florida, Gainesville, FL 32611, USA*

[2] Both authors contributed equally

[3]*Department of Pathology, Immunology, and Laboratory Medicine, University of Florida, Gainesville, FL 32610, USA.*

*Corresponding Author: Dr. Joseph Larkin III

PO Box 110700, Museum Road Building 981

Department of Microbiology and Cell Science

Gainesville, FL 32611

Phone: (352) 392-6884

Fax: (352) 392-5922

E-mail address: jlarkin3@ufl.edu

Supplemental Figure 1: Treatment with the mimetic peptide prevents progression of lymphadenopathy in the MRL/lpr mouse. (A) Scale developed to classify lymphadenopathy presentations (left). Bar graph displaying the percentage of MRL/lpr mice in cohort 1 that had to be euthanized due to severe lymphadenopathy (right). (B) Representative images of lymphadenopathy occurrences.

S1A.

Scale

1 – No Lymphadenopathy

2 – Multiple sites (<2 cm)

3 – Lymph Nodes > 2 cm

4 – Declining health, animal must be euthanized

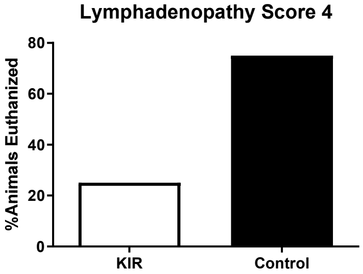

S1B.

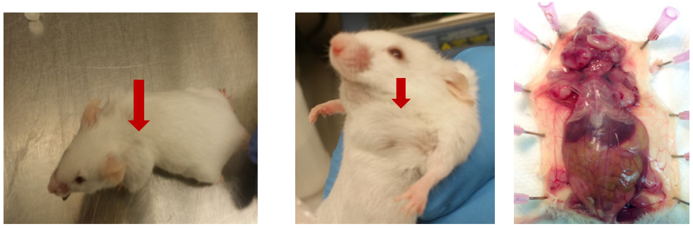

Supplemental Figure 2: Ex vivo T cell activation. Representative flow cytometry data showing the co-expression of CD44 and IFN-γ on PBMCs previously gated on either CD4 or CD8 at 9 and 15 weeks of age.

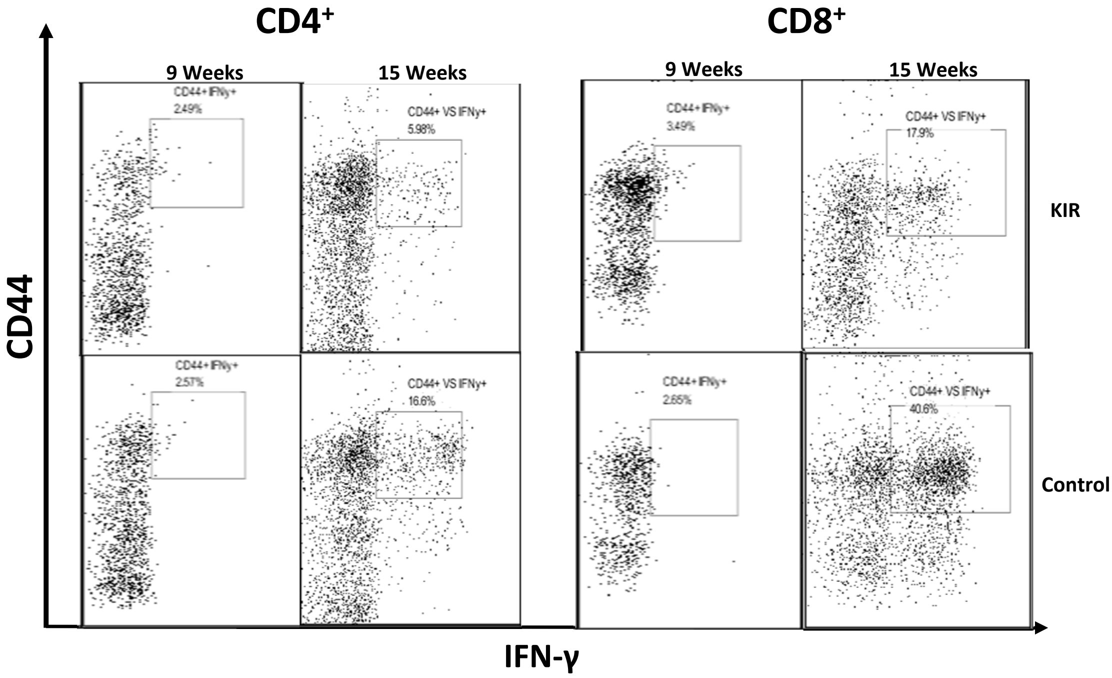

Supplemental Figure 3: Gating strategy for B and T cells. A) Doublet and live/dead discrimination. B) Gating on B cells. C) Gating on CD4+ T regulatory and T follicular regulatory cells. D) Gating on CD4+ and CD8+ populations.

S3A. Doublet and Live/dead discrimination

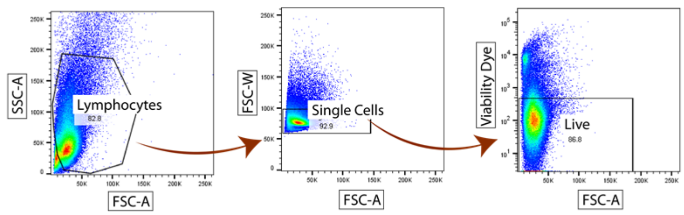

S3B. B cells

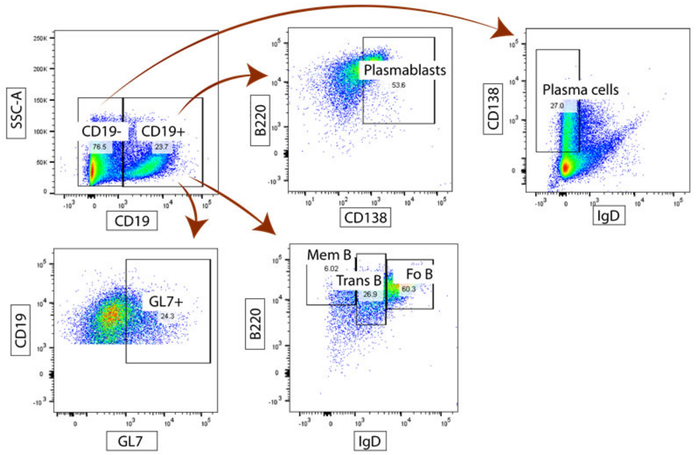

S3C. Fo T and Treg

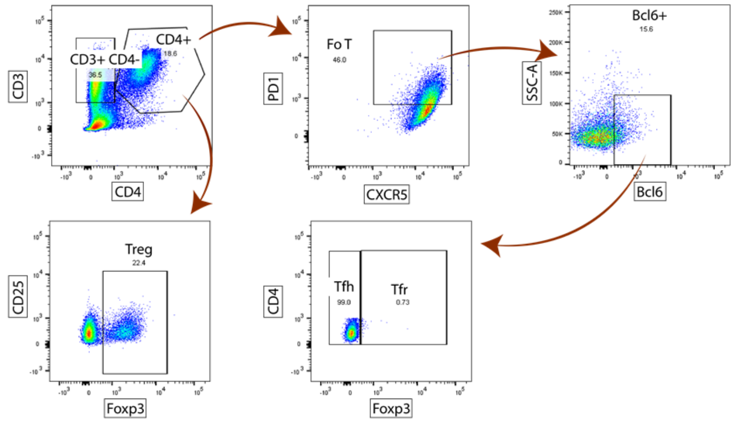

S3D. Tem and Tn

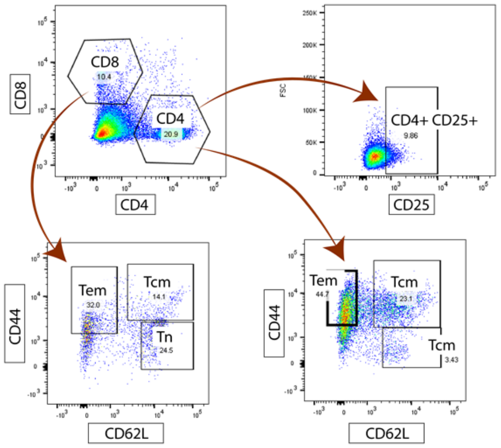

Supplemental Figure 4: T cell populations in the spleen. A) Helper and regulatory follicular T cells. B) Effector and naïve T cell populations were analyzed. *p<0.05, **p<0.01, ***p<0.001 (One-way ANOVA with dunnett's multiple comparison test). Error bars depict SD. Each cohort had 9-10 animals.

S4A.

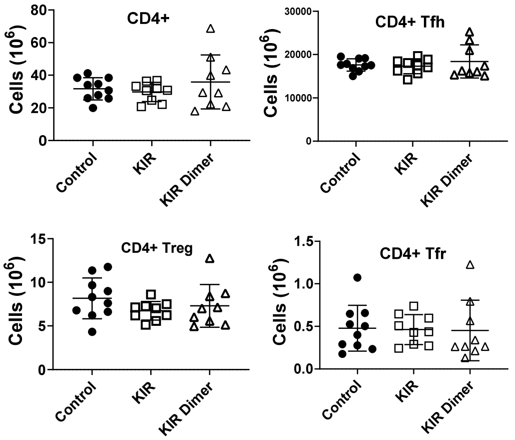

S4B.

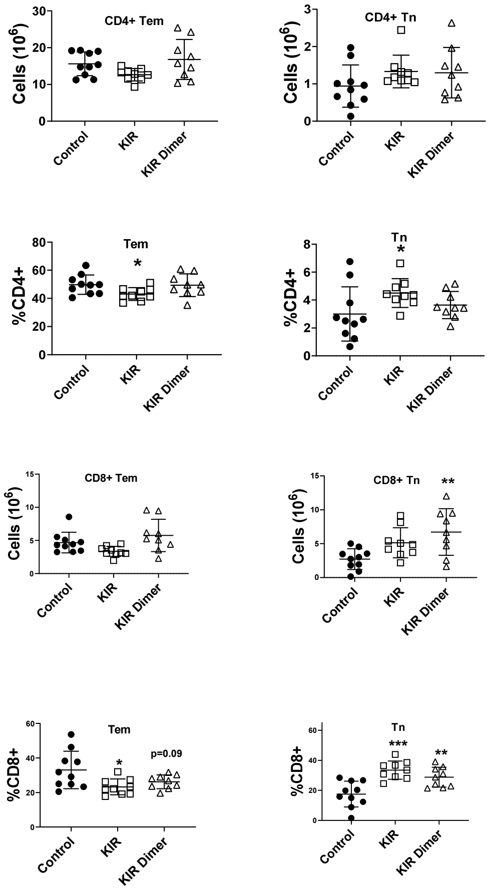

Supplemental Figure 5: B cell populations in the spleen. B cell populations. *p<0.05, **p<0.01, ***p<0.001 (One-way ANOVA with dunnett's multiple comparison test). Error bars depict SD. Each cohort had 9-10 animals.

S5.

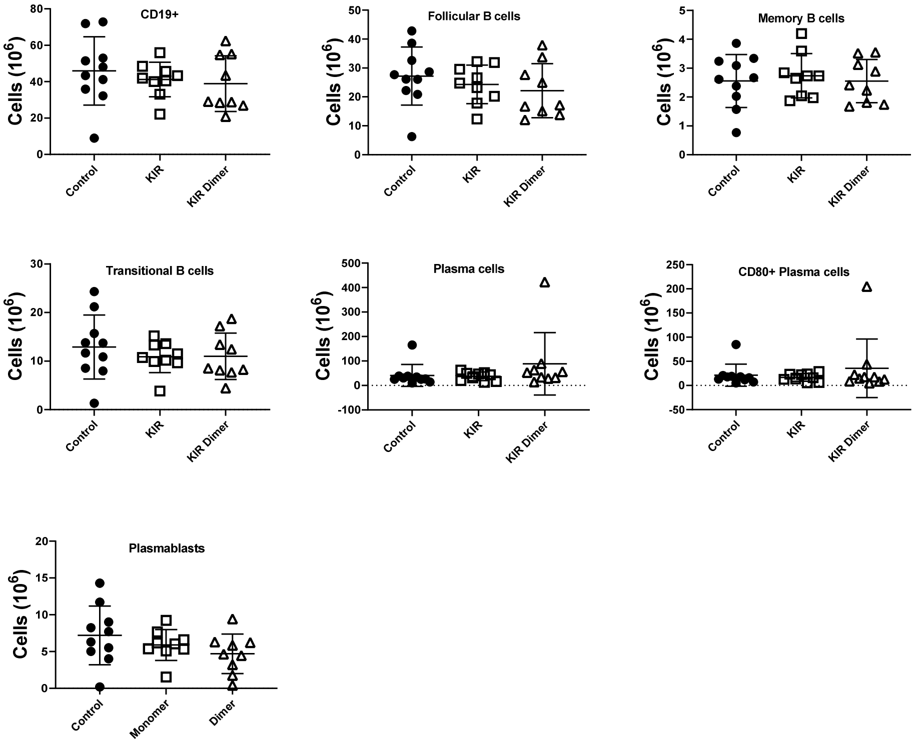

Supplemental Figure 6: ANA correlations. A) Treg Foxp3 MFI vs ANA B) Tfr Foxp3 MFI vs ANA C) Relative frequency of CD4+ naïve T cells vs ANA D) Glomerular area vs ANA. (R) and p values are indicated on the graphs.

S6A.

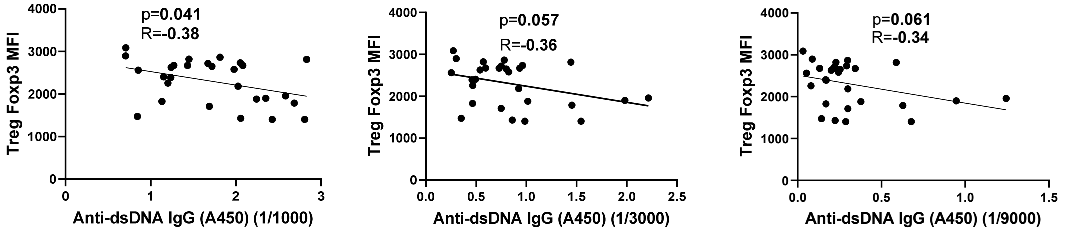

S6B.

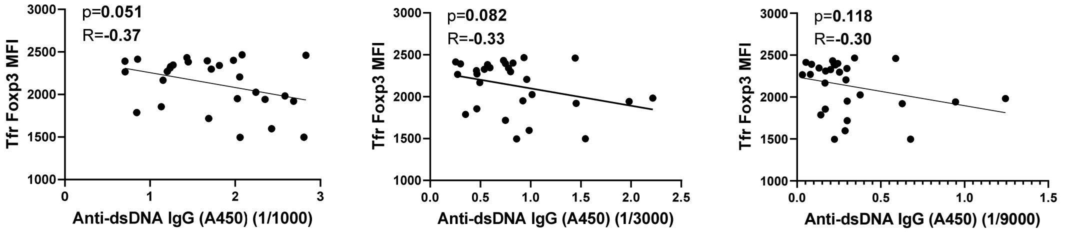

S6C.

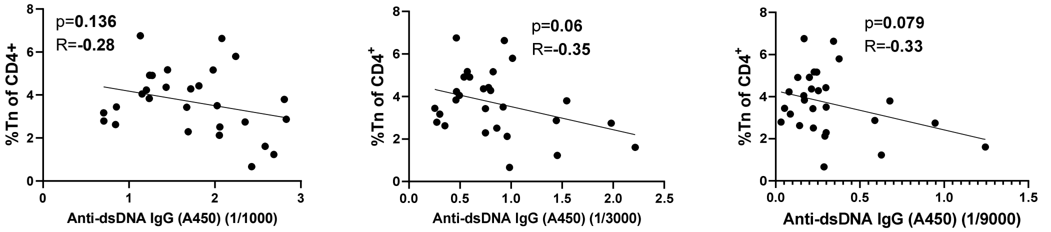

S6D.

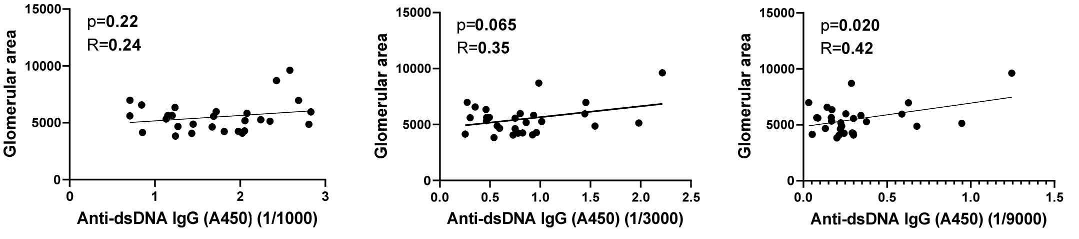

Supplement: Supplementary file 1 — Supplementary information. [file 41598_2021_86017_MOESM1_ESM.pdf]
